# Effects of Psychotherapy on Quality of Life among Patients with Inflammatory Bowel Disease: A Systematic Review with Meta-Analysis

**DOI:** 10.21315/mjms-10-2025-706

**Published:** 2026-04-30

**Authors:** Yashila Subramaniam, Pei Boon Ooi, Min Xi Hoo, Yin Yee Wong, Kelly Phang, Raja Affendi Raja Ali, Roger Pickup

**Affiliations:** 1 School of Psychology, Faculty of Medical and Life Sciences, Sunway University, Bandar Sunway, Selangor, Malaysia; 2 Future Cities Research Institute, Sunway University, Bandar Sunway, Selangor, Malaysia; 3 Faculty of Medicine and Health Sciences, Universiti Putra Malaysia, Serdang, Selangor, Malaysia; 4 Faculty of Health and Medicine, Lancaster University, Lancaster, United Kingdom

**Keywords:** quality of life, inflammatory bowel disease, psychotherapy, good health and well-being, meta-analysis

## Abstract

Individuals diagnosed with inflammatory bowel disease (IBD) have been known to have diminished quality of life (QoL). This present study aimed to examine the impact of psychotherapy on the QoL of patients with IBD. A systematic search for studies was conducted through a total of six databases, namely, MEDLINE, PubMed, Scopus, SAGE, ProQuest (psychology database), and ProQuest (public health database) from their date of inception up to 20 March 2024. The inclusion criteria were studies on patients with IBD, carried out through a randomised controlled trial design, published in the English language, and with full-text content available online. Two researchers independently extracted articles that met the selection criteria and evaluated the quality of each study. A random-effects meta-analysis was conducted, and data were presented in weighted mean difference (WMD) with a 95% CI. Fourteen studies were included in the systematic review, of which three were included in the meta-analysis. Overall, while psychotherapy showed no significant improvements in some QoL domains such as clinical bowel condition and systemic symptoms, notable enhancements were observed in social functioning and overall QoL. The findings underscore the importance of integrating psychotherapeutic approaches into IBD management to address psychological and social challenges, thereby enhancing overall patient well-being. These results have significant implications for clinical mental health counsellors and future research in IBD care.

## Introduction

Inflammatory bowel disease (IBD), which primarily includes Crohn’s disease and ulcerative colitis, is characterised by chronic inflammation of the gastrointestinal tract, leading to symptoms such as abdominal pain, diarrhoea, and fatigue (1). Beyond these physical symptoms, IBD significantly impacts patients’ quality of life (QoL), often contributing to psychological distress, social dysfunction, and reduced life satisfaction (2). Compared to the general population, individuals with IBD experience poorer QoL, higher healthcare costs, and complex treatment needs (3, 4).

QoL is a multifaceted construct encompassing physical health, emotional wellbeing, social engagement, and daily functioning, and is critical for assessing the impact of chronic illnesses like IBD (5). Despite its importance, measuring QoL consistently and in a meaningful way remains challenging due to its subjective and individualised nature. Moreover, QoL assesses the impact of chronic illness and its treatment on individuals’ daily functioning from their perspectives (6). Research has consistently shown that individuals with IBD, both children and adults, report significantly lower QoL than healthy controls (7). The disease and its treatment, including medications and surgeries, can negatively affect patients’ psychological adjustment, academic or occupational performance, and interpersonal relationships (8).

While conventional medical treatments such as medications and surgery are essential for managing the inflammatory aspect of IBD, they do not address the psychological toll of IBD. In recent years, psychotherapy has emerged as a complementary approach to traditional medical care. Psychotherapy encompasses interventions aimed at alleviating psychological distress and enhancing coping strategies, which can play a key role in improving QoL among people with chronic illnesses, including IBD (9, 10). Stress, anxiety, and depression, common comorbidities in IBD, are known to exacerbate disease activity further (9), highlighting the need for psychological support in disease management.

Instruments commonly used to assess QoL in IBD include the Short Form-36 (SF-36) (11) and EuroQoL-5D (EQ-5D) (12), which measure health-related QoL across a range of domains. The Inflammatory Bowel Disease Questionnaire (IBDQ) is the most widely used IBD-specific tool and evaluates QoL across bowel symptoms, systemic symptoms, emotional functioning, and social functioning (13, 14). While individual studies and reviews, such as Mikocka-Walus et al. (9), suggest psychotherapy improves QoL, findings are mixed, and prior reviews are either outdated or focus narrowly on specific interventions or symptomology without quantifying effects across QoL domains.

A variety of psychotherapeutic approaches, including cognitive behavioural therapy (CBT), mindfulness-based stress reduction (MBSR), and psychoeducation, have been applied in the context of IBD. CBT, for example, aims to restructure maladaptive thought patterns and is one of the most frequently studied methods for reducing psychological symptoms in chronic illness populations (9, 15). Similarly, MBSR facilitates present-moment awareness and acceptance of distressing experiences (16), while psychoeducational interventions equip patients with information and coping tools to manage their condition (9).

Therefore, this systematic review and meta-analysis aim to address this gap by synthesising the most recent and relevant evidence on the effects of diverse psychotherapeutic interventions on QoL in individuals with IBD. By quantifying the effect sizes across multiple QoL domains, this study provides evidence-based guidance for integrating psychotherapy into IBD care, particularly in Malaysia, where psychosocial support could enhance patients’ outcomes in resource-limited settings.

## Methods

### Protocol Registration

This review was registered in the PROSPERO (No. CRD42024558564). This review was conducted on the basis of Preferred Reporting Items for Systematic Reviews and Meta-Analyses (PRISMA 2020) guidelines (17).

### Literature Search

One investigator (YS) independently conducted electronic searches for potential studies via MEDLINE, PubMed, Scopus, SAGE Journals, ProQuest (Psychology Database), and ProQuest (Public Health Database) on 20 March 2024. The abovementioned databases were selected to cover medical, psychological, and public health literature, ensuring comprehensive retrieval of studies on psychotherapy and QoL in IBD. The search yielded relevant studies published in the various journals mentioned from inception to 20 March 2024.

### Search Strategies

The search term combinations used were: (psychotherap* OR “cognitive therap*” OR “cognitive psychotherap*” OR “brief psychotherap*” OR “short*term psychotherap*” OR “group psychotherap*” OR “psychodynamic psychotherap*” OR “rational psychotherap*” OR “rational*emotive psychotherap*” OR “imagery psychotherap*” OR “multiple psychotherap*” OR “person-centered psychotherap*” OR “interpersonal psychotherap*” OR “cognitive behavioral psychotherap*” OR “dignity psychotherap*”) AND (“quality of life” OR “health related quality of life” OR “life quality” OR “health-related quality of life” OR “HRQOL”) AND (“inflammatory bowel disease” OR “Crohn’s disease” OR “ulcerative colitis” OR “bowel disease” OR “chronic bowel disease”). The search strategies are presented in Appendix 1.

### Intervention and Control Group Definitions

The term intervention group refers to participants in a study who received any form of psychotherapy during the intervention period. The control group refers to participants who did not receive any form of psychotherapy during the intervention period of the study or after the study.

### Study Screening

Relevant studies identified through the database searches were imported into EndNote 21, reference manager software. Duplicates in the search were removed. Then, the titles and abstracts of the remaining articles were screened based on the search strategies. The full-text articles found were assessed based on the inclusion and exclusion criteria of this study.

The full-text articles found were assessed based on the inclusion criteria mentioned below. This stringent screening procedure ensured that only the most pertinent and methodologically sound papers were included in our analysis. Furthermore, reverse-forward citation tracking was conducted manually from the identified studies. Two authors (MXH and YYW) independently completed each step. Before the complete text of each pertinent article was studied, a discussion was held, and any disagreements between the studies chosen by MXH and YYW were resolved by the senior author (PBO).

### Study Selection

The selection criteria required studies with a randomised controlled trial design on patients with IBD. Only studies that were journal articles, published in English, and had full-text content available were considered suitable. Studies were excluded from the review if they were non-RCTs, unpublished, not in English, or not available in full-text.

### Data Extraction

The following characteristics were extracted from the selected studies: last name of the first author, year of publication, country, sample size, mean ± SD, gender, type of IBD, type of intervention, duration of intervention, information from intervention and control groups (baseline mean ± SD, final mean ± SD, and mean difference ± SD), the *P*-value for difference in mean change between the two groups, and the risk of bias.

### Statistical Analysis

Mean differences in QoL between groups were calculated using either fixed-effects or random-effects meta-analysis, depending on heterogeneity levels. This was carried out using Review Manager 5 (RevMan 5.3) (18), whereby the weighted mean difference (WMD) was subjected to a two-tailed test to yield a statistically significant *P*-value of < 0.05. To assess heterogeneity between the studies, the *I*^2^ index was examined. A fixed-effects model was used for outcomes with low heterogeneity (*I*^2^ < 50%), while a random-effects model was applied for outcomes with high heterogeneity (*I*^2^ > 50%). In addition, publication bias was assessed using funnel plots, Egger’s test, and Begg’s test via Meta-Essentials (19).

## Results

### Quality Assessment

Two authors (YS and MXH) evaluated the risk of bias and methodological quality of each study using the Joanna Briggs Institute (JBI) Critical Appraisal Checklist for Randomised Controlled Trials (RCTs), which comprises 13 items designed to assess the rigour of RCTs (20). This checklist addresses four primary types of bias: bias related to selection and allocation, bias related to the administration of interventions or exposures, bias related to the assessment, detection, and measurement of outcomes, and bias related to participant retention.

The authors independently assessed each study, categorising the items as “yes,” “no,” “unclear,” or “not applicable” to determine the extent to which potential sources of bias were addressed, and any discrepancies were discussed with PBO. Following the risk of bias assessment, data extraction was conducted using JBI’s standardised tools for RCT designs, capturing information on sample size, participant demographics (e.g., age and gender), intervention specifics, study settings and locations, data collection methods, analysis techniques, study designs, data sources, outcome measurements, and key findings. This structured approach ensured a comprehensive synthesis of relevant study characteristics, facilitating meaningful comparisons across studies.

### Description of Included Studies

Figure 1 depicts the literature search and selection process. The literature search initially identified 207 articles. After excluding 71 duplicates, 136 studies were retrieved to review their titles and abstracts. From these, 35 studies were considered eligible to undergo full-text assessment for inclusion criteria. After a comprehensive evaluation of these articles, 14 articles were finally selected for systematic review and three for meta-analysis.

### Characteristic of the Studies

The characteristics of the 14 studies included in this review are summarised and portrayed in Tables 1a and 1b. In total, the subjects of the current systematic review and meta-analysis consisted of 1,457 patients with IBD across the 14 studies. Three studies were conducted in Netherlands (21–23), two in the United Kingdom (UK) (24, 25), two in Australia (26, 27), one each in Brazil (28), Spain (29), Norway (30), the United States of America (USA) (31), New Zealand (32), Scotland (33), and Portugal (34). Out of the 14 papers, only three papers (26, 28, 34) did not report the mean ages of their participants.

The majority of the studies included in this review utilised the IBDQ as the main measure to assess participants’ QoL, except for three papers, which used the IMPACT-III Questionnaire (31) and the IBD Stages of Change Coping Questionnaire (IBDSCCQ) (26, 27).

In terms of therapeutic interventions used in the included studies, five studies applied CBT (21, 24, 26, 27, 31). One study employed transpersonal brief psychotherapy (28), while another used multicomponent CBT (29). Multi-convergent therapy was used in one study (25), and group psychotherapy in another (30). Two studies utilised mindfulness-based cognitive therapy (22, 33), and one study applied mind-body therapy (23). Additionally, computerised CBT was employed in one study (32), while acceptance and commitment therapy was used in another (34).

For meta-analysis, only three studies were included (21, 25, 29). This is because only the studies that reported the mean differences and standard deviations for the IBDQ domain were included in the meta-analysis. Specifically, the mean differences in the four main domains of IBDQ (bowel symptoms, systemic symptoms, social functions, emotional functions) and the overall mean difference in QoL were investigated to obtain a clear picture of QoL.

### Risks of Bias Within Studies

The risk of bias was assessed across five areas: selection and allocation bias, intervention/exposure administration bias, assessment, detection, and outcome measurement bias, and participant retention bias (Table 2c). Some studies did not report mean differences or *P*-values for specific QoL domains, which limited data extraction. Acciari et al. (28) exhibited a high risk of bias due to the lack of random assignment, lack of demographic data, failure to randomise treatment conditions, and the lack of blinding, as the therapists were aware of the treatment conditions. The study by McCombie et al.(32) was rated high risk as the randomisation method was not stated, low adherence rate, and lack of blinding in outcome assessment, with relatively high dropout rates. Lastly, being a study protocol, Trindade et al.’s study was rated high risk due to substantial missing information, including details on participant and assessor blinding and follow-up procedures (34).

In contrast, several other papers that were included in this review had low risk of biases as they included majority of the vital information but left out some details pertaining to the blinding of participants, and randomisation; for example, Bennebroek Evertsz et al. (21) was classified as having a low risk of bias as the study lacked information regarding long-term follow-up. However, all these papers had reliable outcome measures, used appropriate statistical analysis, and included complete follow-up after the period of intervention (22, 23, 25, 30, 31).

Furthermore, the study by Mikocka-Walus et al. (26) was assessed to have a moderate risk, as it had a lack of information on randomisation of the participants but showed appropriate follow-up and accurate outcome measurement. Next, another paper was rated as having a low risk of bias as it had sufficient information on randomisation, reliable outcome measurement tools, and appropriate follow-up (27). A low-risk rating was obtained by Schoultz et al. (33), who had participants unblinded to the assessment but maintained accurate outcome measurement and thorough follow-up because blinding was difficult due to the nature of the intervention (a course).

Overall, nine studies were found to have a low risk of bias (21–25, 27, 30, 31, 33), two studies had a moderate risk (26, 29), and three studies were rated as having a high risk of bias (28, 32, 34).[Table t2a-02mjms3302_ra][Table t2b-02mjms3302_ra]

### Effects of Psychotherapy on QoL and Its Subgroup Analysis

We further examined the effects of psychotherapy on QoL and its subgroups, bowel symptoms, systemic symptoms, social functions, and emotional symptoms. There was no statistically significant improvement in bowel symptoms QoL observed in the intervention group (psychotherapy) compared to the control group (placebo) using a fixed-effects model (WMD = 0.21; 95% CI: −0.05, 0.47), as shown in Table 3. Similarly, no significant improvement in systemic symptoms QoL was found between the intervention and control groups in the fixed-effects model (WMD = 0.14; 95% CI: −0.13, 0.41; see Table 4). The lack of significant improvement in bowel and systemic symptoms suggests that psychotherapy may not directly influence physical disease activity, but its benefits in psychosocial domains warrant further exploration. In the random-effects model for emotional functions QoL, there was also no statistically significant improvement (WMD = 4.30; 95% CI: −1.49, 10.08), as shown in Table 5.

In contrast, a statistically significant improvement in social functions QoL was found in the intervention group compared to the control group using a fixed-effects model (WMD = 0.50; 95% CI: 0.24, 0.76; see Table 6). A significant improvement was also seen in overall QoL outcomes (WMD = 10.84; 95% CI: 5.26, 16.42), as shown in Table 7. The heterogeneity of studies for bowel symptoms, social functions, and overall QoL was low (*I*^2^ = 0%), indicating consistency across studies. However, moderate heterogeneity was observed for systemic symptoms (*I*^2^ = 45%), and high heterogeneity for emotional functions (*I*^2^ = 89%).

### Publication Bias Analysis

The publication bias of each outcome was assessed using funnel plots, Egger’s test, and Begg’s test. Based on the funnel plot for bowel symptoms, Egger’s test (*P* = 0.886) and Begg’s test (*P* = 0.602) suggested that there was no publication bias in the studies. Similarly, the analysis for systemic symptoms suggested no publication bias, as reflected by Egger’s test (*P* = 0.236) and Begg’s test (*P* = 0.117). For social functions, the funnel plot, along with Egger’s test (*P* = 0.244) and Begg’s test (*P* = 0.602), indicated no evidence of publication bias.

The analysis of emotional functions also showed no indication of publication bias, supported by Egger’s test (*P* = 0.715) and Begg’s test (*P* = 0.602). Finally, the funnel plot for the overall QoL indicated no publication bias, with Egger’s test (*P* = 0.887) and Begg’s test (*P* = 0.602). The corresponding funnel plots for each outcome are presented in Figures 2 to 6, which confirmed no publication bias across all domains.

## Discussion

The current systematic review and meta-analysis synthesised evidence from 14 RCTs to investigate the effects of psychotherapy on QoL among patients with IBD. The effectiveness of psychological therapies in the treatment of IBD has been shown to differ. The findings demonstrate that psychotherapeutic interventions, particularly CBT, significantly improve social functioning and overall QoL, though effects on physical domains such as bowel and systemic symptoms are minimal. These results align with prior reviews, such as Mikocka-Walus et al. (9), which reported moderate QoL improvements with psychotherapy, but extend their work by quantifying effect sizes across specific QoL domains using meta-analysis, revealing stronger benefits in psychosocial outcomes.

The meta-analysis, based on the three studies (21, 25, 29), showed significant improvements in social functioning and overall QoL, with low heterogeneity indicating consistent effects across studies. Notably, CBT demonstrated the largest benefits, particularly in Bennebroek Evertsz et al. (21), who reported substantial improvements in social (MD = 0.98) and overall QoL (MD = 15.14). In contrast, no significant improvements were observed in bowel symptoms or systemic symptoms, suggesting that psychotherapy primarily enhances psychosocial rather than physical outcomes. The high heterogeneity in emotional functioning may reflect variations in intervention types (e.g., CBT vs multi-convergent therapy [MCT]) or study populations, such as differences in disease severity or cultural contexts, which warrant further investigation through subgroup analyses.

CBT emerged as the most effective intervention, consistent with its established role in reducing anxiety and depression in chronic illness populations (15, 32). For instance, Bennebroek Evertsz et al. (21) and Artom et al. (24) reported high patient acceptance of CBT, with improvements in psychological distress and social functioning. Mindfulness-based interventions, such as mindfulness-based cognitive therapy (MBCT), also showed potential, particularly for fatigue and psychological symptoms (22, 33). However, other approaches, such as transpersonal brief psychotherapy (28) and acceptance and commitment therapy (34), were less conclusive due to limited sample sizes or methodological limitations. The variability in intervention effectiveness underscores the need for tailored approaches based on patient needs and disease characteristics.

### Clinical Significance of This Review

The significant improvements in social functioning and overall QoL highlight the potential of psychotherapy as an adjunctive treatment in IBD management. The psychological and social burdens brought on by IBD, including impaired relationships and reduced productivity (8), can exacerbate disease activity and healthcare costs (4). By addressing these challenges, psychotherapy, particularly CBT, offers a structured and goal-oriented approach to enhance patient wellbeing (15, 32). In Malaysia and similar emerging healthcare contexts, where access to integrated psychosocial care may vary, these findings may inform the incorporation of psychotherapy as an adjunctive component in multidisciplinary IBD management, although further local evidence is required to substantiate its clinical and cost effectiveness.

The lack of significant effects on physical symptoms suggests that psychotherapy does not directly alter disease activity, consistent with findings from previous research (26, 27). This shows that psychotherapy should complement, rather than replace, conventional treatments like medications or surgery. However, the consistent psychosocial benefits across diverse populations and healthcare systems, as seen in studies from the Netherlands, UK, and Australia (22, 24, 26), highlight the generalizability of psychotherapy as an effective adjunctive intervention for improving patients’ social functioning, emotional well-being, and overall QoL, regardless of geographical and healthcare context.

### Strengths and Limitations

This review’s strength includes its comprehensive inclusion of diverse psychotherapeutic modalities (e.g., CBT, MBCT, MCT) and its focus on RCTs, ensuring methodological rigour. The inclusion of studies from diverse cultural backgrounds enhances the generalizability of findings, particularly for Asian populations where IBD is becoming increasingly prevalent. Lastly, the incorporation of the long-term evaluations of paediatric and adult populations further strengthens the evidence base for psychotherapy’s sustained benefits over time and across age groups (27, 30, 31).

However, limitations must be acknowledged. Firstly, the meta-analysis was constrained by including only three studies, potentially limiting statistical power. The reliance on self-reported QoL measures, such as the IBDQ, introduces potential reporting bias and subjectivity, particularly as these tools may not fully capture cultural nuances in Asian populations, including Malaysia. Inconsistent outcome measurements, small sample sizes in some trials (28), and lack of long-term follow-up in others (29) further limit conclusions. High heterogeneity in emotional functioning outcomes suggests variability in intervention delivery or patient characteristics, which could not be fully explored due to limited data.

### Future Directions

Future research should prioritise standardised psychotherapy protocols to reduce heterogeneity and enhance comparability across studies. Longer follow-up periods are essential to assess the durability of QoL improvements, particularly for emotional and social domains. Developing culturally adapted QoL measures, such as the IBDQ version tailored for Malaysian or Asian patients, could improve outcome assessment accuracy. Additionally, exploring the cost-effectiveness of psychotherapy in resource-limited settings could potentially inform healthcare policy in Malaysia and similar contexts.

## Conclusion

This systematic review and meta-analysis provide preliminary evidence that psychotherapeutic intervention, particularly CBT, significantly enhances social functioning and overall QoL in patients with IBD, despite the limited effects on physical symptoms. These findings support the integration of psychotherapy as an adjunctive treatment in comprehensive IBD care, addressing psychosocial challenges that exacerbate disease burden. In Malaysia, where the prevalence of IBD is rising, incorporating CBT into clinical practice could improve patient well-being, especially in resource-limited settings. In order to optimise outcomes, future research should focus on standardised intervention protocols, culturally adapted QoL measures, and long-term follow-up studies to ensure applicability across diverse populations, including Malaysia. These advancements will strengthen evidence-based management and enhance patient-centred care globally.

## Figures and Tables

**Figure 1:**
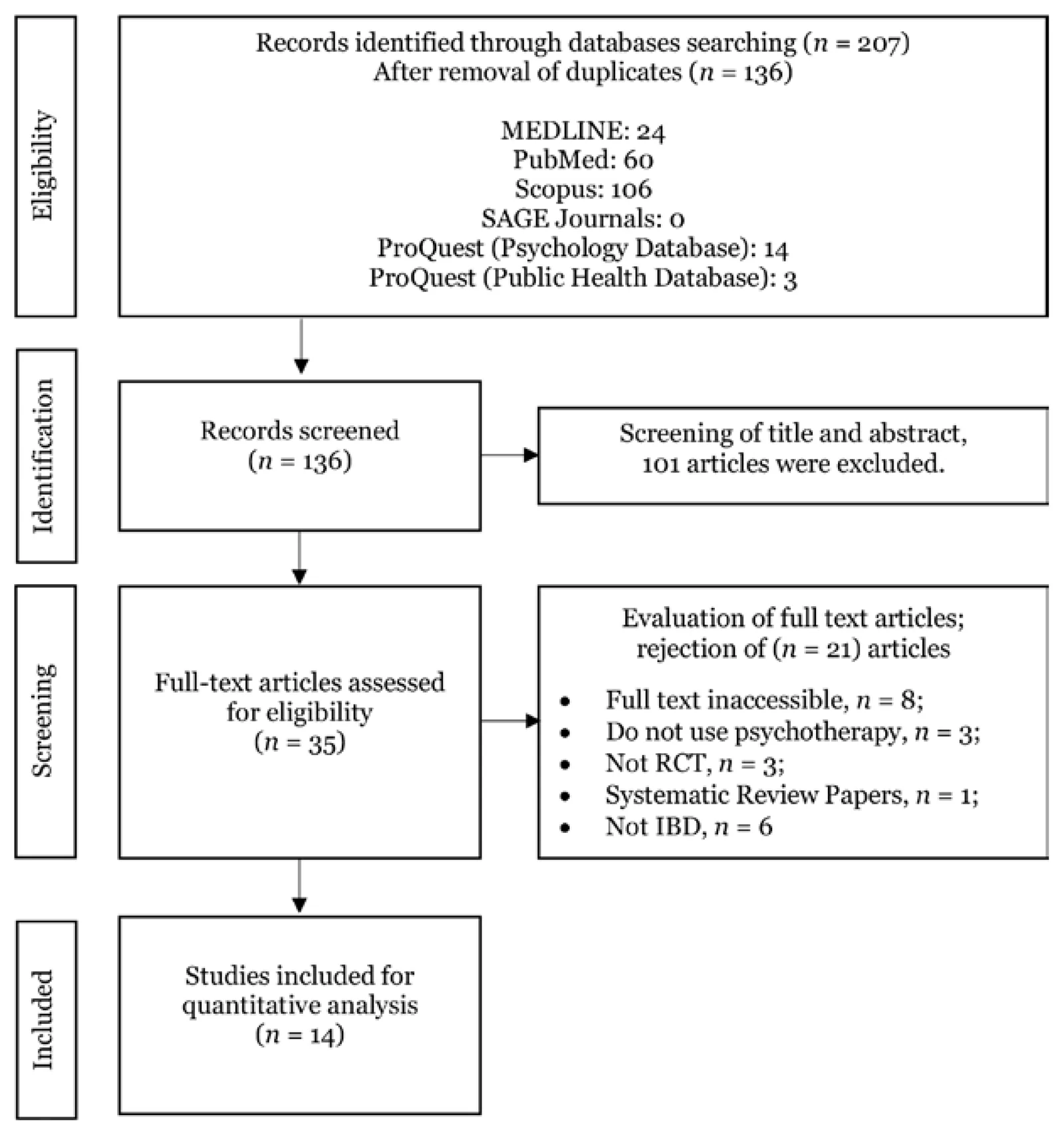
The PRISMA diagram details our search and selection process applied during the overview

**Figure 2:**
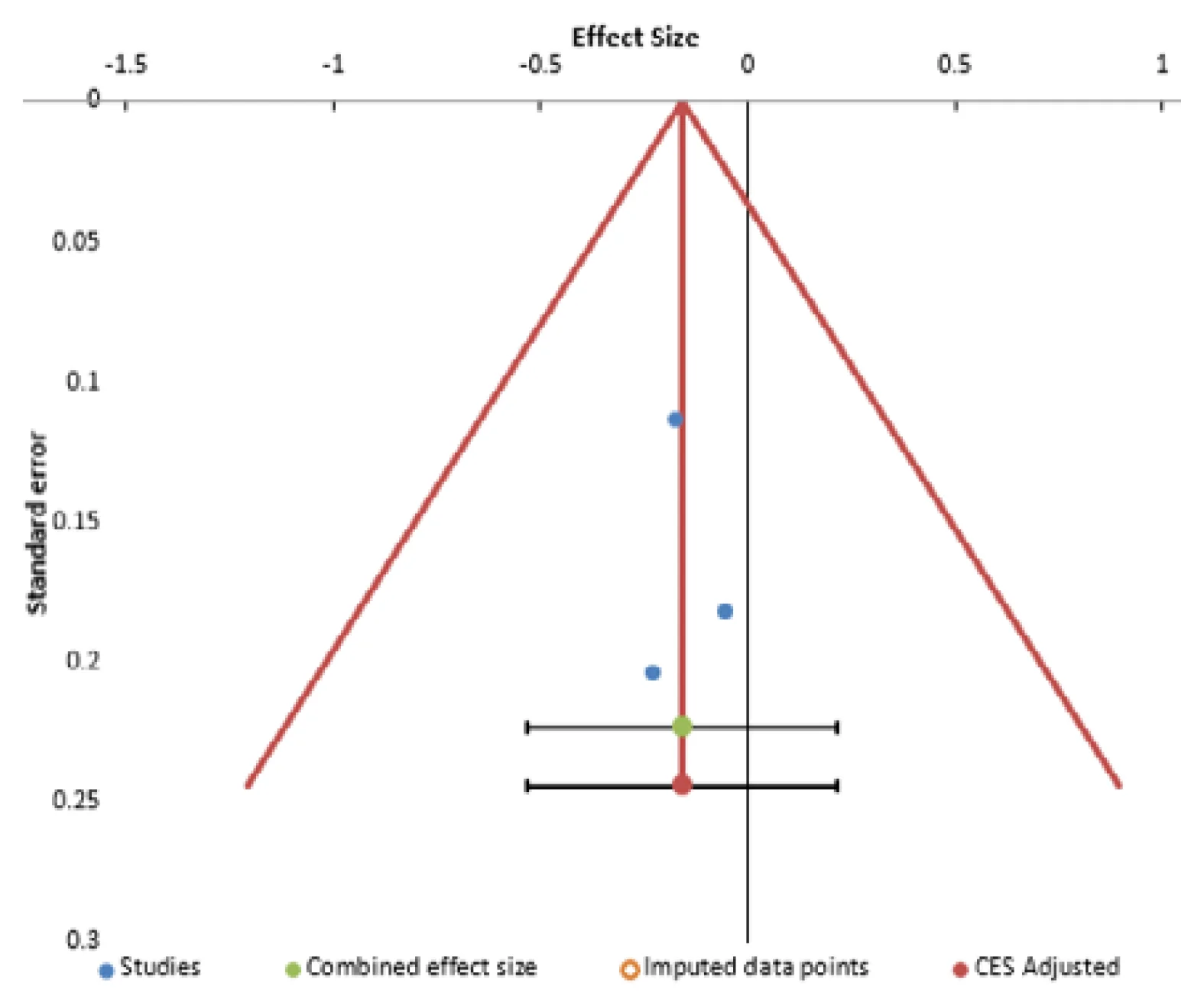
Funnel plot of studies evaluating the effects of psychotherapy on QoL (bowel symptoms) in IBD patients

**Figure 3:**
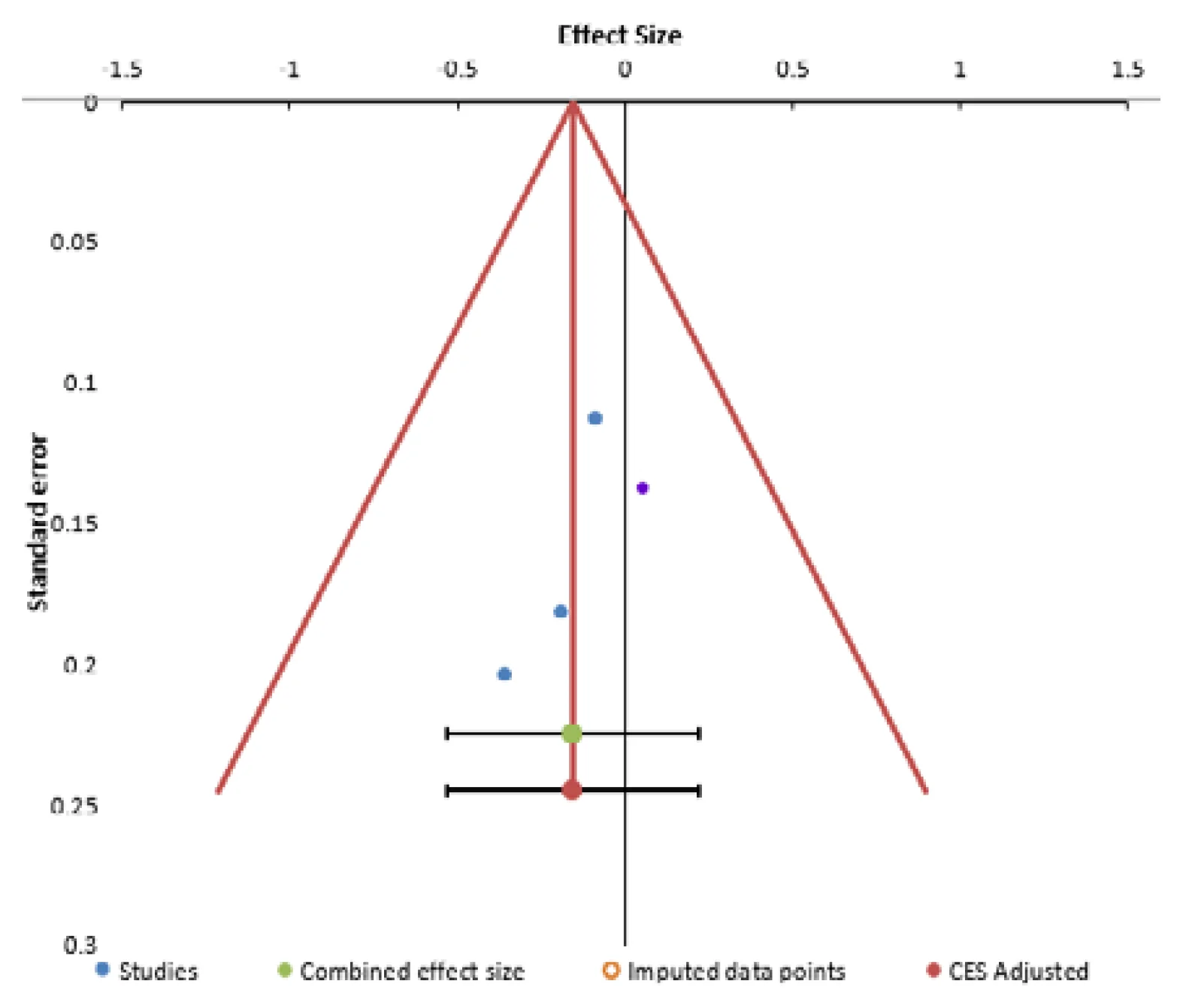
Funnel plot of studies evaluating the effects of psychotherapy on QoL (systemic symptoms) in IBD patients

**Figure 4:**
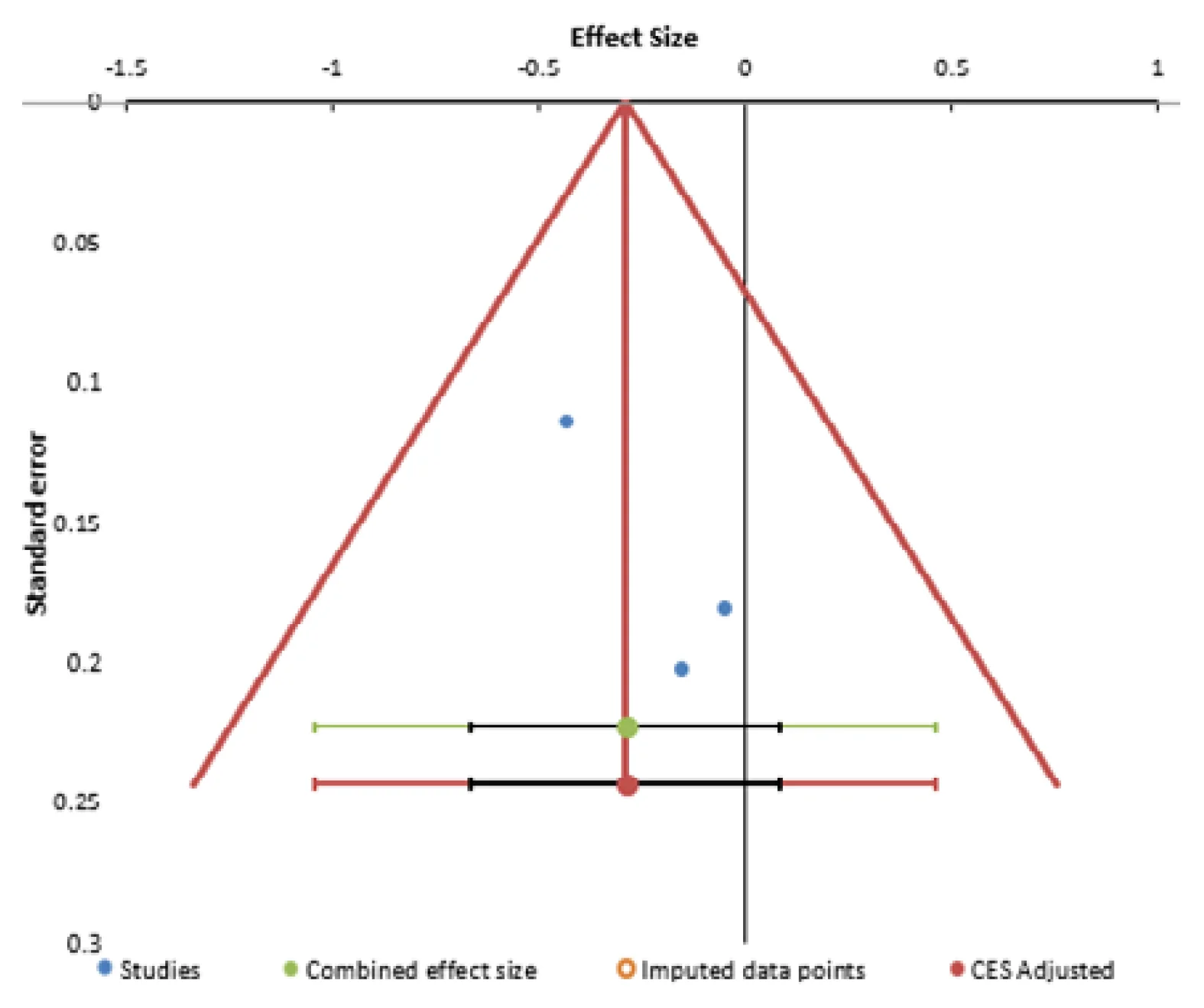
Funnel plot of studies evaluating the effects of psychotherapy on QoL (social functions) in IBD patients

**Figure 5:**
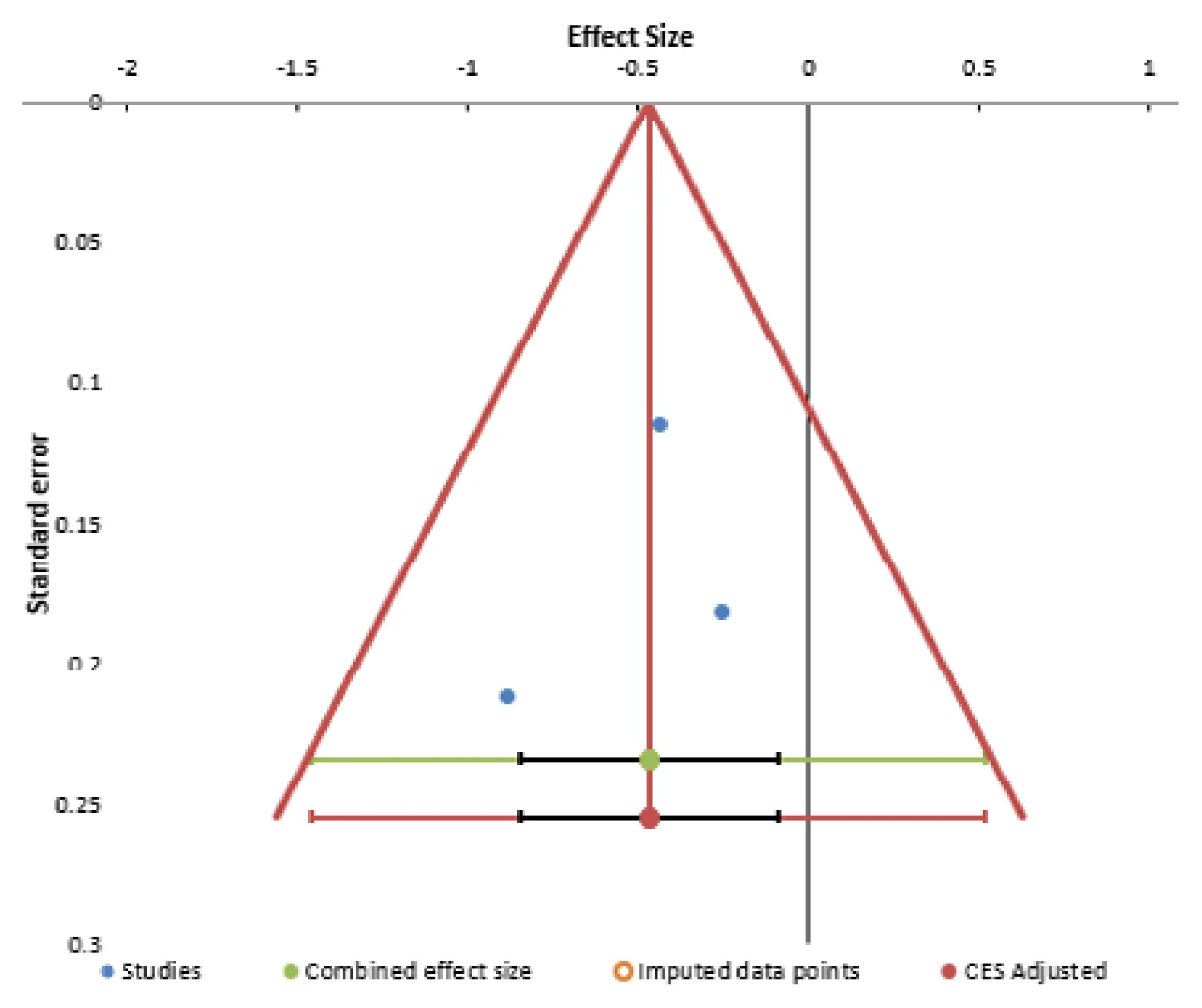
Funnel plot of studies evaluating the effects of psychotherapy on QoL (emotional functions) in IBD patients

**Figure 6:**
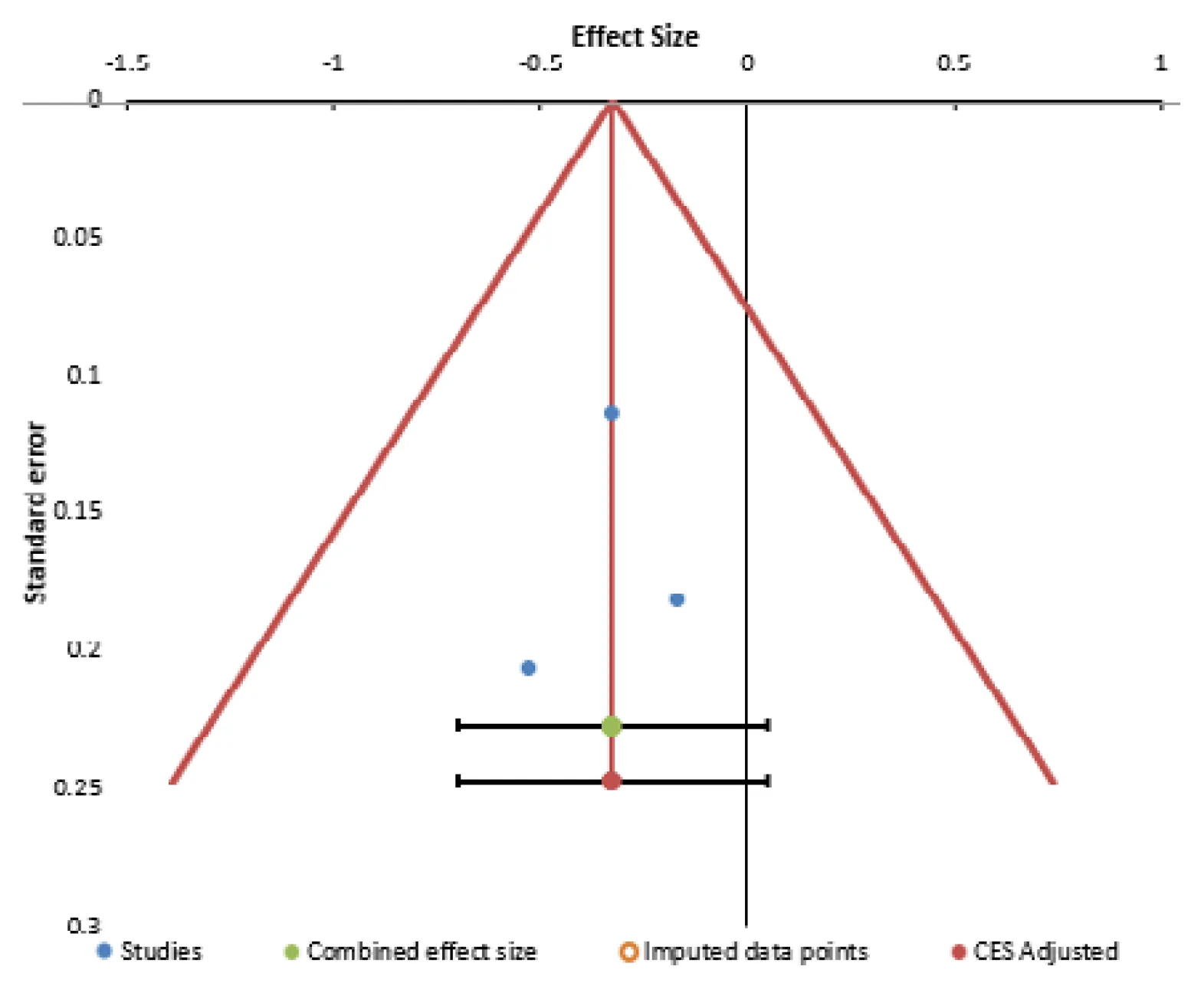
Funnel plot of studies evaluating the effects of psychotherapy on QoL (overall QoL) in IBD patients

**Table 1a:** Demographic characteristics of the studies included in the Systematic Review

Author	Country	Sample size	Mean age ± SD (Intervention)	Mean age ± SD (Control)	Gender (M/F)
Acciari et al. (28)	Brazil	11	N/A	N/A	N/A
Artom et al. (24)	UK	31	37.00 ± N/A	39.13 ± N/A	11/20
Bennebroek Evertsz’ et al. (21)	Netherlands	118	39.40 ± N/A	38.70 ± N/A	75/43
Bernabeu et al. (29)	Spain	120	44.50 ± 11.81	42.00 ± 11.65	47/73
Berrill et al. (25)	UK	66	44.40 ± N/A	45.40 ± N/A	15/51
Boye et al., 2011 (30)	Norway	114	40.40 ± 11.00	36.80 ± 9.60	78/36
Bredero et al. (22)	Netherlands	113	47.30 ± 12.70	46.0 ± 14.80	43/70
Elsenburch et al. (23)	Netherlands	30	42.90 ± 8.60	42.40 ± 11.40	10/20
Levy et al. (31)	USA	185	44.50 ± 6.60	43.80 ± 7.10	18/167
McCombie et al. (32)	New Zealand	199	39.60 ± N/A	38.30 ± N/A	71/128
Mikocka-Walus et al. (26)	Australia	174	N/A	N/A	N/A
Mikocka-Walus et al. (27)	Australia	176	46.50 ± 15.70	51.90 ± 16.90	94/82
Schoultz et al. (33)	Scotland	44	48.59 ± 15.37	49.68 ± 15.37	10/34
Trindade et al. (34)	Portugal	76	N/A	N/A	N/A

**Table 1b:** Intervention characteristics of the studies included in the Systematic Review

Author	Type of intervention	Duration of intervention	Measure/s of QoL
Acciari et al. (28)	Transpersonal brief psychotherapy	N/A	IBDQ
Artom et al. (24)	Cognitive behavioural therapy	8 weeks	IBDQ
Bennebroek Evertsz’ et al. (21)	Cognitive behavioural therapy	3.5 months	IBDQ
Bernabeu et al. (29)	Multicomponent cognitive behavioural therapy	8 weeks	IBDQ
Berrill et al. (25)	Multi-convergent therapy	4 months	IBDQ
Boye et al. (30)	Group psychotherapy	3 months	IBDQ
Bredero et al. (22)	Mindfulness-based cognitive therapy	8 weeks	IBDQ
Elsenburch et al. (23)	Mind-body therapy	10 weeks	IBDQ
Levy et al. (31)	Cognitive behavioural therapy	6 months	IMPACT-III Questionnaire
McCombie et al. (32)	Computerised cognitive behavioural therapy	12 weeks	IBDQ
Mikocka-Walus et al. (26)	Cognitive behavioural therapy	10 weeks	IBDSCCQ
Mikocka-Walus et al. (27)	Cognitive behavioural therapy	10 weeks	IBDSCCQ
Schoultz et al. (33)	Mindfulness-based cognitive therapy	8 weeks	IBDQ
Trindade et al. (34)	Acceptance and commitment therapy	N/A	IBDQ

QoL = Quality of Life; SDQ; IBDQ = Inflammatory Bowel Disease Questionnaire; IBDSCCQ = IBD Stages of Change Coping Questionnaire

**Table 2a t2a-02mjms3302_ra:** QoL outcomes for bowel and systemic symptoms in intervention and control groups

Author	QoL (bowel symptoms) (Mean difference ± SD / *P*-value)		QoL (systemic symptoms) (Mean difference ± SD / *P*-value)	
	Intervention	Control	Intervention	Control
Acciari et al. (28)	12.6 ± 6.7 / P = 0.001	N/A	8.6 ± 5.5 / *P* = 0.001	N/A

Artom et al. (24)	N/A	N/A	N/A	N/A

Bennebroek Evertsz’ et al. (21)	*P* = 0.364	*P* = 0.155	*P* = 0.053	*P* = 0.595

Bernabeu et al. (29)	N/A	N/A	N/A	N/A

Berrill et al. (25)	N/A	N/A	N/A	N/A

Boye et al. (30)	N/A	N/A	N/A	N/A

Bredero et al. (22)	N/A	N/A	N/A	N/A

Elsenburch et al. (23)	N/A	N/A	N/A	N/A

Levy et al. (31)	N/A	N/A	N/A	N/A

McCombie et al. (32)	N/A	N/A	N/A	N/A

Mikocka-Walus et al. (26)	N/A	N/A	N/A	N/A

Mikocka-Walus et al. (27)	N/A	N/A	N/A	N/A

Schoultz et al. (33)	N/A	N/A	N/A	N/A

Trindade et al. (34)	N/A	N/A	N/A	N/A

**Table 2b t2b-02mjms3302_ra:** QoL outcomes for social and emotional functions in intervention and control groups

Author	QoL (social functions) (Mean difference ± SD / *P*-value)		QoL (emotional functions) (Mean difference ± SD / *P*-value)	
	Intervention	Control	Intervention	Control
Acciari et al. (28)	7.4 ± 4.6 / *P* = 0.001	N/A	5.0 ± 2.8 / *P* = 0.001	N/A

Artom et al. (24)	N/A	N/A	N/A	N/A

Bennebroek Evertsz’ et al. (21)	*P* = 0.00	*P* = 0.357	*P* = 0.00	*P* = 0.103

Bernabeu et al. (29)	N/A	N/A	N/A	N/A

Berrill et al. (25)	N/A	N/A	N/A	N/A

Boye et al. (30)	N/A	N/A	N/A	N/A

Bredero et al. (22)	N/A	N/A	N/A	N/A

Elsenburch et al. (23)	N/A	N/A	N/A	N/A

Levy et al. (31)	N/A	N/A	N/A	N/A

McCombie et al. (32)	N/A	N/A	N/A	N/A

Mikocka-Walus et al. (26)	N/A	N/A	N/A	N/A

Mikocka-Walus et al. (27)	N/A	N/A	N/A	N/A

Schoultz et al. (33)	N/A	N/A	N/A	N/A

Trindade et al. (34)	N/A	N/A	N/A	N/A

**Table 2c:** Overall QoL outcomes and risk of bias assessment for included studies

Author	QoL (overall)		Risk of bias
	Intervention group (Mean difference ± SD / *P*-value)	Control group (Mean difference ± SD / *P*-value)	
Acciari et al. (28)	33.6 ± 13.4 / *P* = 0.001	N/A	High risk

Artom et al. (24)	N/A	N/A	Low risk

Bennebroek Evertsz’ et al. (21)	*P* = 0.001	*P* = 0.149	Low risk

Bernabeu et al. (29)	N/A	N/A	Moderate risk

Berrill et al. (25)	N/A	N/A	Low risk

Boye et al. (30)	N/A	N/A	Low risk

Bredero et al. (22)	N/A	N/A	Low risk

Elsenburch et al. (23)	N/A	N/A	Low risk

Levy et al. (31)	N/A	N/A	Low risk

McCombie et al. (32)	N/A	N/A	High risk

Mikocka-Walus et al. (26)	N/A	N/A	Moderate risk

Mikocka-Walus et al. (27)	N/A	N/A	Low risk

Schoultz et al. (33)	N/A	N/A	Low risk

Trindade et al. (34)	N/A	N/A	High risk

**Table 3:** Forest plot of the effects of psychotherapy on QoL (bowel symptoms) among IBD patients

Study	Psychotherapy			Control			Weight (%)	Mean difference IV, fixed, 95% CI
	Mean	SD	Total	Mean	SD	Total		
Berrill et al. (25)	5.40	1.10	167	5.20	1.2	145	99.1	0.20 (−0.06, 0.46)

Bernabeu et al. (29)	55.70	10.20	60	55.10	12.3	60	0.4	0.60 (−3.44, 4.64)

Bennebroek Evertsz’ et al. (21)	53.63	9.07	49	51.45	9.8	47	0.5	2.18 (−.60, 5.96)

Total (95% CI)			276			252	100.0	0.21 (−0.05, 0.47)

Heterogeneity: Chi^2^ = 1.08; *df* = 2 (*P* = 0.58); *I*^2^ = 0%; Test for overall effect: *Z* =1.61 (*P* = 0.11)

**Table 4:** Forest plot of the effects of psychotherapy on QoL (systemic symptoms) among IBD patients

Study	Psychotherapy			Control			Weight (%)	Mean difference IV, fixed, 95% CI
	Mean	SD	Total	Mean	SD	Total		
Berrill et al. (25)	4.3	1.0	167	4.20	1.40	145	97.5	0.10 (−0.17, 0.37)

Bernabeu et al. (29)	24.3	6.0	60	23.10	6.80	60	1.4	1.20 (−1.09, 3.49)

Bennebroek Evertsz’ et al. (21)	22.2	6.2	49	19.89	6.69	47	1.1	2.31 (−0.27, 4.89)

Total (95% CI)			276			252	100.0	0.14 (−0.13, 0.41)

Heterogeneity: Chi^2^ = 3.61, *df* = 2 (*P* = 0.16); *I*^2^ = 45%; Test for overall effect: *Z* =1.01 (*P* = 0.31)

**Table 5:** Forest plot of the effects of psychotherapy on QoL (emotional functions) among IBD patients

Study	Psychotherapy			Control			Weight (%)	Mean difference IV, fixed, 95% CI
	Mean	SD	Total	Mean	SD	Total		
Berrill et al. (25)	5.0	1.00	167	4.50	1.30	145	38.3	0.50 (0.24, 0.76)

Bernabeu et al. (29)	66.2	10.20	60	62.70	16.40	60	30.1	3.50 (−1.39, 8.39)

Bennebroek Evertsz’ et al. (21)	64.8	11.36	49	55.13	10.35	47	31.5	9.67 (5.33, 14.01)

Total (95% CI)			276			252	100.0	4.30 (−1.49, 10.08)

Heterogeneity: Tau^2^ = 22.70; Chi^2^ = 18.47, *df* = 2 (*P* < 0.0001); *I*^2^ = 89%; Test for overall effect: *Z* =1.46 (*P* = 0.15)

**Table 6:** Forest plot of the effects of psychotherapy on QoL (social functions) among IBD patients

Study	Psychotherapy			Control			Weight (%)	Mean difference IV, fixed, 95% CI
	Mean	SD	Total	Mean	SD	Total		
Berrill et al. (25)	6.20	0.90	167	5.70	1.40	145	97.5	0.50 (0.23, 0.77)

Bernabeu et al (29)	29.90	5.90	60	29.60	6.50	60	1.4	0.30 (−1.92, 2.52)

Bennebroek Evertsz’ et al. (21)	27.49	6.49	49	26.51	6.11	47	1.1	0.98 (0.23, 3.50)

Total (95% CI)			276			252	100.0	0.50 (0.24, 0.76)

Heterogeneity: Chi^2^ = 0.17, *df* = 2 (*P* = 0.92); *I*^2^ = 0%; Test for overall effect: *Z* =3.75 (*P* = 0.0002)

**Table 7:** Forest plot of the effects of psychotherapy on QoL (overall) among IBD patients

Study	Psychotherapy			Control			Weight (%)	Mean difference IV, fixed, 95% CI
	Mean	SD	Total	Mean	SD	Total		
Berrill et al. (25)	167.0	30.0	167	156.00	37.00	145	54.7	11.00 (3.45, 18.55)

Bernabeu et al. (29)	176.2	28.0	60	170.60	38.70	60	21.3	5.60 (−6.49, 17.69)

Bennebroek Evertsz’ et al. (21)	168.2	28.6	49	152.98	28.35	47	24.0	15.14 (3.75, 26.53)

Total (95% CI)			276			252	100.0	10.84 (5.26, 16.42)

Heterogeneity: Chi^2^ = 1.27, *df* = 2 (*P* = 0.53); *I*^2^ = 0%; test for overall effect: *Z* = 3.81 (*P* = 0.0001)

## Appendix

**Appendix 1:** Search terms used for final search on 20 March 2024

Searches	Search terms	MedLine	PubMed	Scopus	SAGE	ProQuest (Psychology Database)	ProQuest (Public Health Database)
#1	psychotherap* OR “cognitive therap*” OR “cognitive psychotherap*” OR “brief psychotherap*” OR “short*term psychotherap*” OR “group psychotherap*” OR “psychodynamic psychotherap*” OR “rational psychotherap*” OR “rational*emotive psychotherap*” OR “imagery psychotherap*” OR “multiple psychotherap*” OR “interpersonal psychotherap*” OR “cognitive behavioral psychotherap*” OR “dignity psychotherap*”	105,258	59,241	220,439	519	28,156	6,359
#2	“quality of life” OR “health related quality of life” OR “life quality” OR “health-related quality life” OR “HRQOL”	472, 649	396,340	738,662	2,801	42,424	74,767
#3	“inflammatory bowel disease” OR “Crohn’s disease” OR “ulcerative colitis” OR “bowel disease” OR “chronic bowel disease”	120, 369	120,668	233	751	2,152	7,537
#4	1 AND 2 AND 3	25	60	106	0	16	4
#5	Limit #4 to peer reviewed articles and English	24				14	3

## References

[1] Danese S, Fiocchi C (2011). Ulcerative colitis. N Engl J Med.

[2] Narula N, Dhillon A, Zhang D, Sherlock ME, Tondeur M, Zachos M (2018). Enteral nutritional therapy for induction of remission in Crohn’s disease. Cochrane Database Syst Rev.

[3] Gracie DJ, Hamlin PJ, Ford AC (2019). The influence of the brain–gut axis in inflammatory bowel disease and possible implications for treatment. Lancet Gastroenterol Hepatol.

[4] Parra RS, Chebli JMF, Amarante H, Flores C, Parente JML, Ramos O (2019). Quality of life, work productivity impairment and healthcare resources in inflammatory bowel diseases in Brazil. World J Gastroenterol.

[5] Kolovos S, Kleiboer A, Cuijpers P (2016). Effect of psychotherapy for depression on quality of life: meta-analysis. Br J Psychiatry.

[6] Irvine EJ (2008). Quality of life of patients with ulcerative colitis: past, present, and future. Inflamm Bowel Dis.

[7] Knowles SR, Graff LA, Wilding H, Hewitt C, Keefer L, Mikocka-Walus A (2018). Quality of life in inflammatory bowel disease: a systematic review and meta-analyses—part I. Inflamm Bowel Dis.

[8] Matos R, Lencastre L, Rocha V, Torres S, Vieira F, Barbosa MR (2021). Quality of life in patients with inflammatory bowel disease: the role of positive psychological factors. Health Psychol Behav Med.

[9] Mikocka-Walus A, Pittet V, Rossel JB, von Känel R (2016). Symptoms of depression and anxiety are independently associated with clinical recurrence of inflammatory bowel disease. Clin Gastroenterol Hepatol.

[10] Phang K, Abdul Latif AAL, Lee KW, Ching SM, Ooi PB (2022). Effects of psychotherapy on quality of life in end-stage renal disease patients: a systematic review with meta-analysis. Oman Med J.

[11] Lins L, Carvalho FM (2016). SF-36 total score as a single measure of health-related quality of life: scoping review. SAGE Open Med.

[12] König HH, Ulshöfer A, Gregor M, von Tirpitz C, Reinshagen M, Adler G (2002). Validation of the EuroQol questionnaire in patients with inflammatory bowel disease. Eur J Gastroenterol Hepatol.

[13] Sexton KA, Walker JR, Targownik LE, Graff LA, Haviva C, Beatie BE (2019). The inflammatory bowel disease symptom inventory: a patient-report scale for research and clinical application. Inflamm Bowel Dis.

[14] Cheung WY, Garratt AM, Russell IT, Williams JG (2000). The UK IBDQ—a British version of the inflammatory bowel disease questionnaire: development and validation. J Clin Epidemiol.

[15] van Straten A, Geraedts A, Verdonck-de Leeuw I, Andersson G, Cuijpers P (2010). Psychological treatment of depressive symptoms in patients with medical disorders: a meta-analysis. J Psychosom Res.

[16] Segal ZV, Dimidjian S, Beck A, Boggs JM, Vanderkruik R, Metcalf CA (2020). Outcomes of online mindfulness-based cognitive therapy for patients with residual depressive symptoms: a randomized clinical trial. JAMA Psychiatry.

[17] Liberati A, Altman DG, Tetzlaff J, Mulrow C, Gøtzsche PC, Ioannidis JPA (2009). The PRISMA statement for reporting systematic reviews and meta-analyses of studies that evaluate health care interventions: explanation and elaboration. J Clin Epidemiol.

[18] (2014). Review Manager (RevMan) [computer program]. Version 5.3.

[19] Suurmond R, van Rhee H, Hak T (2017). Introduction, comparison, and validation of Meta-Essentials: a free and simple tool for meta-analysis. Res Synth Methods.

[20] Barker TH, Stone JC, Sears K, Klugar M, Leonardi-Bee J, Tufanaru C (2023). Revising the JBI quantitative critical appraisal tools to improve their applicability: an overview of methods and the development process. JBI Evid Synth.

[21] Bennebroek Evertsz F, Sprangers MAG, Sitnikova K, Stokkers PCF, Ponsioen CY, Bartelsman J (2017). Effectiveness of cognitive-behavioral therapy on quality of life, anxiety, and depressive symptoms among patients with inflammatory bowel disease: a multicenter randomized controlled trial. J Consult Clin Psychol.

[22] Bredero QM, Fleer J, Smink A, Kuiken G, Potjewijd J, Laroy M (2023). Mindfulness-based cognitive therapy for fatigue in patients with inflammatory bowel disease: results of a randomized controlled trial. Mindfulness (NY).

[23] Elsenbruch S, Langhorst J, Popkirowa K, Müller T, Luedtke R, Franken U (2005). Effects of mind-body therapy on quality of life and neuroendocrine and cellular immune functions in patients with ulcerative colitis. Psychother Psychosom.

[24] Artom M, Czuber-Dochan W, Sturt J, Proudfoot H, Roberts D, Norton C (2019). Cognitive-behavioural therapy for the management of inflammatory bowel disease–fatigue: a feasibility randomized controlled trial. Pilot Feasibility Stud.

[25] Berrill JW, Sadlier M, Hood K, Green JT (2014). Mindfulness-based therapy for inflammatory bowel disease patients with functional abdominal symptoms or high perceived stress levels. J Crohns Colitis.

[26] Mikocka-Walus A, Bampton P, Hetzel D, Hughes P, Esterman A, Andrews JM (2015). Cognitive-behavioural therapy has no effect on disease activity but improves quality of life in subgroups of patients with inflammatory bowel disease: a pilot randomized controlled trial. BMC Gastroenterol.

[27] Mikocka-Walus A, Bampton P, Hetzel D, Hughes P, Esterman A, Andrews JM (2017). Cognitive-behavioural therapy for inflammatory bowel disease: 24-month data from a randomised controlled trial. Int J Behav Med.

[28] Acciari A, Leão F, Coy C, Leal R, Dias C, Ayrizono M (2015). Effects of transpersonal brief psychotherapy on general state of health and life quality of patients with Crohn’s disease. J Coloproctol.

[29] Bernabeu P, van-der Hofstadt C, Rodríguez-Marín J, Gutierrez A, Alonso MR, Zapater P (2021). Effectiveness of a multicomponent group psychological intervention program in patients with inflammatory bowel disease: a randomized trial. Int J Environ Res Public Health.

[30] Boye B, Lundin KE, Jantschek G, Leganger S, Mokleby K, Tangen T (2011). INSPIRE study: does stress management improve the course of inflammatory bowel disease and disease-specific quality of life in distressed patients with ulcerative colitis or Crohn’s disease? A randomized controlled trial. Inflamm Bowel Dis.

[31] Levy RL, van Tilburg MA, Langer SL, Romano JM, Walker LS, Mancl LA (2016). Effects of a cognitive behavioral therapy intervention trial to improve disease outcomes in children with inflammatory bowel disease. Inflamm Bowel Dis.

[32] McCombie A, Gearry R, Andrews J, Mulder R, Mikocka-Walus A (2016). Does computerized cognitive behavioral therapy help people with inflammatory bowel disease? A randomized controlled trial. Inflamm Bowel Dis.

[33] Schoultz M, Atherton I, Watson A (2015). Mindfulness-based cognitive therapy for inflammatory bowel disease patients: findings from an exploratory pilot randomized controlled trial. Trials.

[34] Trindade IA, Pereira J, Galhardo A, Ferreira NB, Lucena-Santos P, Carvalho SA (2021). The LIFEwithIBD intervention: study protocol for a randomized controlled trial of a face-to-face acceptance and commitment therapy and compassion-based intervention tailored to people with inflammatory bowel disease. Front Psychiatry.

